# Hematopoietic stem cell transplantation from HLA-matched sibling donors in children with acute lymphoblastic leukemia: A report from the Children’s Cancer Hospital Egypt

**DOI:** 10.3389/fonc.2022.983220

**Published:** 2022-09-27

**Authors:** Mahmoud Hammad, Hanafy Hafez, Iman Sidhom, Dina Yassin, Sherine Salem, Khaled Alsheshtawi, Nayera Hamdy, Nahla Elsharkawy, Alaa Elhaddad

**Affiliations:** ^1^ Paediatric Oncology Department National Cancer Institute, Cairo University and Children’s Cancer Hospital Egypt, Cairo, Egypt; ^2^ Clinical Pathology Department National Cancer Institute, Cairo University and Children’s Cancer Hospital Egypt, Cairo, Egypt; ^3^ Clinical Research Department Children’s Cancer Hospital Egypt, Cairo, Egypt

**Keywords:** matched sibling donor, hematopoietic stem cell transplantation, acute lymphoblastic leukemia, pediatric, low and middle-income countries

## Abstract

**Introduction:**

Allogeneic hematopoietic stem cell transplantation (HSCT) is widely used for high-risk acute lymphoblastic leukemia (ALL) patients in their first complete remission (CR1), and for relapsed patients in second complete remission (CR2).

**Patients and methods:**

We retrospectively analyzed data for 67 children with ALL, from a cancer center in a low/middle income country, who had undergone HSCT from human leukocyte antigen (HLA)-matched sibling donors (MSDs) using myeloablative conditioning (MAC) regimens, between 2007 and 2020, describing the survival outcome and relapse probability after achieving CR1 and CR2 and determining outcome differences in relation to indications for HSCT in patients transplanted in CR1. All patients had achieved a negative minimal residual disease prior to transplant (<0.01%).

**Results:**

Forty-six patients (68.7%) were in CR1; 25 had adverse cytogenetics, including 18 patients with Philadelphia chromosome-positive ALL (Ph-positive ALL), and 21 had poor induction response. The 5-year overall survival (OS), event-free survival (EFS) and cumulative incidence of relapse (CIR) for the whole cohort were 56.1% (95% CI, 42.8%-69.4%), 49% (95% CI, 35.7%-62.3%) and 33.5% (95% CI, 21.7%-45.8%), respectively with better EFS and CIR for CR1 transplants compared to CR2 transplants (P=0.02 and P=0.03, respectively). Patients with Ph-positive ALL had better 5-year OS, EFS and non-relapse mortality (NRM) compared with other CR1 transplants (P=0.015, P=0.009 and P=0.028, respectively).

**Conclusion:**

Hematopoietic stem cell transplantation from MSD for ALL in CR1 group had superior outcomes compared to CR2 group and was apparently a curable option for Ph-positive ALL without an increased risk of non-relapse mortality. Poorer survival rates and higher relapse probabilities were associated with HSCT conducted to patients who had a poor response to induction therapy or suffered a relapse.

## Introduction

The outcome of pediatric acute lymphoblastic leukemia (ALL) has improved markedly over the last five decades, with 5-year event-free survival (EFS) now exceeds 80%. Allogeneic hematopoietic stem cell transplantation (HSCT) remains the only curative option for certain subgroups of ALL patients in their first complete remission (CR1) to prevent disease relapse, as well as to treat patients who had relapsed and achieved a second complete remission (CR2) (1, 2). Indications of HSCT in childhood ALL in CR1 are continuously being revised as advances are made in molecular sequencing, chemotherapy and immunotherapy (1, 3).

Induction failure (IF) and high minimal residual disease (MRD), detected at the end of induction or consolidation, are indicators of poor prognosis. In such cases, considerably better survival is achieved with HSCT compared with chemotherapy alone (4, 5).

Poor outcome has been reported in patients with hypodiploid karyotype (< 44 chromosomes), with an 8-year EFS of only 38.5%. The exact indications for HSCT in CR1 for patients with hypodiploid karyotype are yet to be delineated. Current preference is to reserve HSCT for patients with positive MRD at the end of induction (6, 7).

Stratifying treatment for children with hypodiploid ALL based on MRD level was coupled with improved survival outcome, while allogeneic transplantation compared with chemotherapy alone did not reflect on improved outcome, especially among patients who achieved a negative MRD at end of induction therapy with an adjusted 5-year disease-free survival rate (DFS) of 59.8% and 53.0% (P = .47), respectively (8).

Historically, Philadelphia chromosome-positive ALL (Ph-positive ALL) was one of the most unfavorable subtypes of ALL, with a median survival of less than a year (9). Results of the Children’s Oncology Group (COG) and other ALL trials have demonstrated improved survival rates in patients who had received intensive chemotherapy plus a tyrosine kinase inhibitor (TKI) compared with those who had received chemotherapy alone. Similar EFS rates are now being reported for patients who had undergone HSCT in CR1 compared with those who had received chemotherapy plus imatinib (10, 11). The current prevailing practice is to base the decision to conduct HSCT for Ph-positive ALL patients in CR1 on the depth of remission using an MRD response assessment along with combined cytogenetics or molecular testing for BCR/ABL1 (10, 12, 13).

An improved leukemia-free survival and overall survival (OS) were observed in patients who had relapsed and underwent an HLA-matched HSCT in CR2 compared to those who received chemotherapy only (14).

Results of two BFM studies reported comparable survival outcomes and non-relapse mortality (NRM) among pediatric patients transplanted in CR1 or CR2, with no discernable differences in disease-free survival between recipients of grafts from HLA-matched sibling donors (MSDs) and matched unrelated donors (MUDs), with EFS rates of 65% to 67% (15, 16).

We aimed to describe the outcomes (OS, EFS and NRM) and relapse probabilities in a group of pediatric ALL patients who had undergone HSCT in CR1 or CR2 from MSDs using myeloablative conditioning (MAC) regimens. We further aimed to determine outcome differences in relation to indications for HSCT in patients transplanted in CR1.

## Material and methods

This retrospective study included pediatric ALL patients (≤18 years of age at diagnosis) who had undergone allogeneic HSCT at the Children’s Cancer Hospital between August 2007 and December 2020 and were followed up to May 2021. Patients had been initially treated according to the St. Jude Total-XV protocol (17). Eligibility criteria for HSCT at our center is detailed in **Table 1**. Patients had undergone HSCT according to the availability of MSD and remission status at time of transplant (MRD <0.01). Hypodiploidy ALL and Ph-positive ALL were considered high-risk features and eligible to undergo HSCT in CR1 according to our center policy until 2018 and regardless of their MRD response at end of induction.

**Table 1:** Indications of allogeneic HSCT in first and second complete remission for the whole ALL cohort from HLA-MSD (as per data collected).

Disease status	Indications
ALL-CR1	− Hypodiploid ALL* − Ph-positive ALL* − Infantile ALL with t (4,11)(q21;q23) − Induction failure (>5% blasts in the bone marrow) − MRD ≥1% at end of induction
ALL-CR2	− Early bone marrow relapse: <36 months from diagnosis − Early isolated extramedullary: <18 months from diagnosis − Late relapse (B-precursor ALL) with positive MRD at the end of reinduction − Any T-cell ALL

*Patients with a matched sibling donor were eligible to undergo a hematopoietic stem cell transplant in first complete remission (CR1) regardless the minimal residual disease response until end of 2018.

ALL, acute lymphoblastic leukemia; CR1, first complete remission; CR2, second complete remission; HLA-MSD, human leukocyte antigen-matched sibling donor; HSCT, hematopoietic stem cell transplant; MRD, minimal residual disease; Ph-positive, Philadelphia chromosome-positive.

### Pretransplant assessment

Response assessment was conducted using a six-color flow cytometry assay for immunophenotyping. Additional molecular monitoring with qPCR for BCR/ABL1 fusion gene was routinely used for Ph-positive ALL. All included patients in this study had achieved a negative MRD by immunophenotyping (<0.01%) prior to transplant.

Human leukocyte antigen-typing (HLA-typing) was determined using intermediate resolution and 7/8 to 8/8 HLA-matched sibling donors were selected. Bone marrow stem cells (BMSCs) were the main graft source.

Pretransplant assessment, performed within 4 weeks prior to HSCT, included bone marrow studies (morphology, cytogenetics and flow cytometry) and a cerebrospinal fluid analysis. For all patients, pre-established donor/recipient-specific polymorphisms were used for post-HSCT donor chimerism studies. Assessment was repeated on days +30 and +100 post-HSCT to screen for early relapse.

### Conditioning and graft versus host disease prophylaxis

A MAC regimen, essentially comprised of total body irradiation (TBI; 1200 cGy) and cyclophosphamide (CTX; 2 × 60 mg/kg), was given for the majority of patients (79%). Busulfan-based conditioning was given for 14 patients including 2 infants. Cyclosporine (CSA)/short courses of methotrexate (MTX) were used as graft-versus-host disease (GVHD) prophylaxis for 80.6% of patients. Since 2019, anti-thymocyte globulin (ATG, Fresenius; 10 mg/kg/dose on days -3 to -1) or posttransplant cyclophosphamide (PTC) have been additionally administered to 11 (16.4%) patients who had received peripheral blood stem cells (PBSCs).

Patients who had suffered central nervous system (CNS) relapse before HSCT received monthly intrathecal triple therapy (MTX, hydrocortisone, and cytarabine) for one-year post-HSCT. For Ph-positive ALL, imatinib was given for one-year post-HSCT.

### Definitions

Event-free survival (EFS) was defined as the time from the date of HSCT to the date of the first event (relapse or death) or to last follow-up. Overall survival (OS) was defined as the time from the date of HSCT to the date of death or of the last follow-up. Transplant related mortality (TRM) is defined as death from any cause in the first 28 days post-HSCT irrespective of disease status. Non-relapse mortality (NRM) was defined as death without the competing event of relapse following HSCT. Absolute neutrophilic count (ANC) engraftment was defined as an ANC of >0.5x10^9^/L (first of 3 consecutive days). Platelet engraftment was defined as a platelet count of >20x10^9^/L (first of 7 consecutive days without prior transfusions).

### Statistical analysis

Data were described using frequencies and percentages for categorical variables or means and standard deviations (medians and range) for numerical variables. Differences between non-normally distributed variables were tested using the Mann-Whitney U test. We performed a logistic regression analysis to estimate the odds ratios and 95% confidence intervals (CIs). Overall survival and EFS were estimated using the Kaplan Meier’s method and estimates were compared by log rank test. The cumulative incidence of relapse (CIR) and CI-NRM were assessed using Gray’s method. Differences in cumulative incidences were examined using the Fine and Gray competing risk regression model. Analysis was performed at level of significance of 0.05.

## Results

### Patients’ characteristics

Sixty-seven pediatric ALL patients were included in this study. Of those, 68.7% were transplanted in CR1, and 31.3% in CR2. The median age of patients at diagnosis was 8 years (range, 0.35-17.7 years) with 62.6% of patients being ≤ 10 years of age. More than 50% of patients younger than 10 years (26/42, 56.5%) were transplanted in CR1. Forty-six (68.7%) were male patients. Forty-nine patients (73%) were B-cell ALL. Among patients who were transplanted in CR1, 25 (54.3%) patients had adverse cytogenetics, including 18 patients with Ph-positive ALL, 6 with hypodiploid karyotype (< 44 chromosomes) and one infantile ALL with t(4, 11). Twenty-one patients had poor response to induction therapy, 10 had induction failure and 11 had high MRD level at end of induction. More than 75% of patients transplanted in CR2 had an early relapse ≤36 months from initial diagnosis, and 23.8% had a late relapse and high MRD at end of reinduction. The median time to relapse before HSCT was 26 months (range, 2.4 – 66.4 month). CNS involvement was detected in 11 patients (**Table 2**).

**Table 2:** Clinical and biological characteristics of the 67 ALL patients.

Characteristics	n (%)
**All patients**	67 (100)
**Gender**	
Male	46 (68.7)
Female	21 (31.3)
**Age at diagnosis, Median (range)**	8 years (0.35-17.7)
**Age at diagnosis**	
≤ 1 year	3 (4.5)
> 1- ≤10 years	39 (58.2)
> 10 years	25 (37.3)
**Immunophenotype**	
T-cell	18 (26.9)
B-cell	49 (73.1)
**Disease status at transplant**	
CR1	46 (68.7)
CR2	21 (31.3)
**Indications for HSCT in CR1**	
Ph-positive ALL; t(9;22)	18 (39.1)
Hypodiploidy (<44 chromosome)	6 (13.0)
t(4;11)	1 (2.2)
Poor induction response	21 (45.7)
**Time to first pre-HSCT relapse (for CR2)**	
≤18 months	5 (23.8)
>18-≤36 months	11 (52.4)
>36 months	5 (23.8)
**Site of pre-HSCT relapse (for CR2)**	
Bone Marrow	10 (47.6)
Isolated CNS	7 (33.3)
Combined	4 (19)

ALL, acute lymphoblastic leukemia CNS, central nervous system; CR1, first complete remission; CR2, second complete remission; HSCT, hematopoietic stem cell transplantation; Ph-positive, Philadelphia chromosome-positive.

### Allogeneic-HSCT procedure

The overwhelming majority of patients (97%) had fully HLA-MSDs, and 2 had one antigen mismatch. Donor and recipient sex were matched in 49.3% of patients. Bone marrow was more frequently used as a stem cell source in 61.2% of patients and the rest received PBSCs mobilized by granulocyte colony-stimulating factor (GCSF). For GVHD prophylaxis, 54 patients received CSA and MTX. Eight patients additionally received ATG (two received BMSCs from one-Ag mismatched sibling donor, and 6 received PBSCs from fully MSDs). Posttransplant cyclophosphamide was given to another 5 patients who were transplanted from PBSCs (**Table 3**).

**Table 3:** Transplant details and outcomes of the 67 ALL patients.

Characteristics	n (%)
**All patients**	67 (100)
**Conditioning regimen**	
TBI based	53 (79)
Non-TBI based (Bu-based)	14 (21)
**Graft Source**	
Bone Marrow	42 (62.7)
Peripheral blood	25 (37.3)
**Donor/recipient sex**	
F to F	10 (15)
F to M	23 (34.3)
M to F	11(16.4)
M to M	23 (34.3)
**Donor/recipient CMV serological status**	
Positive/positive	32 (47.8)
Positive/negative	9 (13.4)
Negative/negative	7 (10.4)
Negative/positive	19 (28.4)
**GVHD prophylaxis**	
CSA + MTX	54 (80.6)
CSA + MTX + ATG	8 (11.9)
CSA + MTX + PTC	5 (7.5)
**Acute GVHD**	19 (28.3)
Grade I-II	9 (13.4)
Grade III-IV	10 (14.9)
**Chronic GVHD (cGVHD)**	17 (25.4)
**Maximum overall severity of cGVHD**	
Mild	3 (4.5)
Moderate	7 (10.4)
Severe	7 (10.4)
**Number of cGVHD organ involved**	
Single	5 (7.4)
Multiple	12 (18)
**Early complications post-HSCT**	
Sinusoidal obstruction syndrome	2 (3)
Capillary leak	4 (6)
Engraftment syndrome	8 (12)
Hemorrhagic cystitis	11 (16.4)
**Relapse post-HCST**	20 (29.9)
For CR1 patients	10/46 (21.7)
For CR2 patients	10/21 (47.6)
**Non-relapse mortality**	10 (14.9)
**Survival Status**	
Alive	42 (62.7)
Dead	25 (37.3)

ALL, acute lymphoblastic leukemia; ATG, Anti-thymocyte globulin; BU-based, busulfan-based; cGVHD, chronic graft versus host disease; CR1, first complete remission; CR2, second complete remission; CSA, cyclosporine; F, female; GVHD, graft versus host disease; HSCT, hematopoietic stem cell transplantation; M, male; MTX, methotrexate; PTC; posttransplant cyclophosphamide; TBI, total body irradiation.

All patients were successfully engrafted with a median time to ANC engraftment of 15 days (range, 9-77 days), and a median time to platelet engraftment of 23.5 days (range, 12-100 days). Platelet engraftment was significantly faster in patients transplanted in CR1 compared with CR2 (21 and 27 days, P=0.004) and in patients who had received PBSCs compared with BMSCs (19 and 24 days, 0.017). Time to ANC engraftment did not statistically differ in relation to disease status at HSCT (P=0.36) or the source of stem cells (P=0.127).

### Early HSCT toxicities and GVHD

Of the entire cohort, 2 patients were diagnosed with sinusoidal obstruction syndrome, 2 had capillary leak syndrome and 11 patients developed hemorrhagic cystitis with no documented transplant related mortality. Nineteen (28.3%) patients experienced acute GVHD (aGVHD) of whom 13 were in CR1 (**Table 3**). Grade III-IV aGVHD occurred in 10 of 19 patients (52.6%), with 7 had ≥ 2 organs involved, lower gastrointestinal tract was involved in all of them, and 3 had isolated skin. The risk of aGVHD was not different between patients transplanted in CR1 and CR2 (p=0.6), but a higher risk of aGVHD was associated with BM transplants than PB transplants (OR, 4.987; 95% CI, 1.28-19.3; p=0.02). Furthermore, a lower risk of aGVHD was observed in transplanted patients ≤12 years of age (OR, 0.28; 95% CI, 0.094-0.841; p=0.02). With regards to GVHD prophylaxis, aGVHD occurred in 18/54 (33.3%) patients who had received CSA and MTX alone and in 1/5 patients who had additionally received PTC prophylaxis while no aGVHD was noted in patients who had additionally received ATG.

Chronic GVHD occurred in 25.3% of patients. Maximum overall severity of cGVHD was mild, moderate and severe in 3, 7 and 7 patients, respectively. Skin was the commonest organ involved in patients with cGVHD (12/17 patients, 70.5%) and presented as a single organ in 3 patients or combined with other organs in 9 patients (**Table 3**).

There were no discernable differences in the risk of developing cGVHD in relationship to age of patients at transplant, conditioning regimens, source of stem cells and remission status before transplant. It is worth mentioning that cGVHD was more common in patients who had received PBSCs plus CSA/MTX (42%) compared with those who had received ATG or PTC in addition to CSA/MTX (0%). The impact of transplant variables on the occurrence of GVHD is detailed in **Supplementary Tables 1, 2**.

### Survival outcome

**Figure 1:**
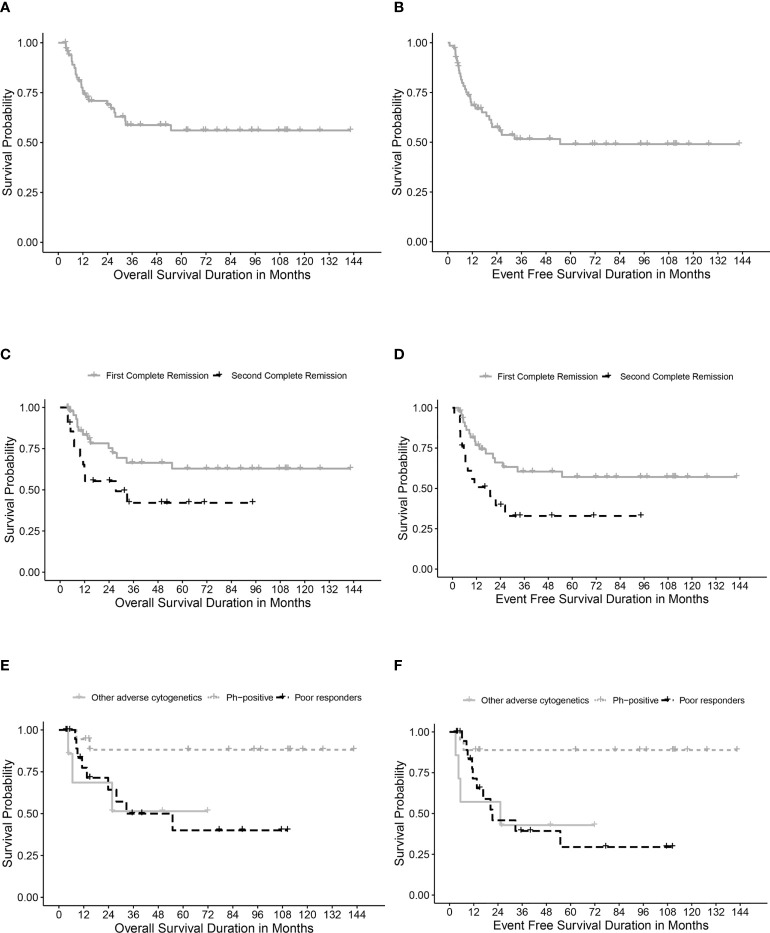
Survival rates. **(A)** Overall survival (OS) for the whole cohort. **(B)** Event-free survival (EFS) for the whole cohort. **(C)** OS for transplanted patients in first (CR1) and second (CR2) complete remission. **(D)** EFS for transplanted patients in first (CR1) and second (CR2) complete remission. **(E)** OS in relation to high-risk features in CR1 transplants. **(F)** EFS in relation to high-risk features in CR1 transplants.

**Table 4** describes different pretransplant characteristics in relationship to transplant outcomes. The 5-year OS and EFS for the whole cohort were 56.1% (95% CI, 42.8%-69.4%) and 49% (95% CI, 35.7%-62.3%), respectively (**Figures 1A, B** **)**. Statistically significantly better EFS was observed in CR1 transplants compared with CR2 (57.1%, 95% CI, 41.2%-73%, and 32.9, 95% CI, 11.1%-54.7%, respectively, P=0.021), while OS was better in CR1 transplants with no statistical significance, P=0.059 (**Figures 1C, D** **)**. Among those transplanted in CR1, Ph-positive ALL had the best 5-year OS (88.1%, 95% CI, 72.6-100%) and EFS (88.9%, 95% CI, 74.4%-100%) compared with other high-risk patients in the CR1 transplant group (P=0.015 for OS and 0.006 for EFS) (**Figures 1E, F** **)**.

**Table 4:** Survival outcomes (OS and EFS), cumulative incidence of relapse (CIR) and cumulative incidence of non-relapse mortality (CI-NRM) post-HSCT in CR1 and CR2 transplants.

Characteristics	5-year OS				5-year EFS				5-year CIR				5-year CI-NRM			
	%	(Confidence Interval)		P	%	(Confidence Interval)		P	%	(Confidence Interval)		P	%	(Confidence Interval)		P
		Lower	Upper			Lower	Upper			Lower	Upper			Lower	Upper	
**Total Patients (N=67)**	56.1	42.8	69.4		49	35.7	62.3		33.6	21.7	45.8		17.4	8.7	28.6	
**Disease status**				0.05				**0.02**				**0.03**				0.94
CR1 (n=46)	62.9	47	78.8		57.1	41.2	73		24.9	12.6	39.2		18.1	7.7	32	
CR2 (n=21)	42.1	19	65.2		32.9	11.1	54.7		52.2	26.5	72.7		14.9	3.5	34.1	
**CR1 subgroup**				**0.02**				**0.009**				0.25				**0.02**
Poor induction response (n=21)	40	13.3	66.7		29.5	5	54		31.2	10.5	54.7		39.4	13.5	64.8	
Ph-positive ALL (n=18)	88.1	72.6	100		88.9	74.4	100		11.1	1.74	30.4		0	0	0	
Other adverse cytogenetics (n=7)*	51.4	11.4	91.4		42.9	6.2	79.6		42.9	7.2	76.2		14.3	0.45	49.6	
**CR2 subgroup**				0.52				0.51				0.62				0.71
CNS relapse (isolated/combined) (n=11)	34.1	4.7	63.5		22.7	0	49.4		59.1	19.7	84.3		18.2	2.4	46	
BM relapse (n=10)	54	20.1	87.9		43.8	9.5	78.1		46.3	10.4	76.9		10	0.4	37.6	
**Graft source**				0.41				0.53				0.40				0.75
BM (n=42)	54.4	38.5	70.3		47.7	32	63.4		37.9	25.3	56.8		14.4	6.9	30.1	
PB (n=25)	61.3	38.5	84		54.4	31.7	77.1		23.9	10.9	52.2		21.7	9.3	51	
**Conditioning regimen**				0.42				0.18				0.10				0.90
TBI based (n=53)	60.6	46.1	75.1		54.6	39.7	69.5		28	17.6	44.8		17.4	9.2	32.9	
Non TBI (n=14)	43.3	15.3	71.3		32.1	6.6	57.6		52.2	31.5	88.2		15.2	4.3	53.6	
**Age at diagnosis**				0.21				0.08				**0.007**				0.20
≤ 10 years (n=42)	50.8	33.6	68		42.7	26.2	59.2		46.8	32.9	66.8		10.4	4.1	26.3	
> 10 years (n=25)	64.4	44	84.8		59.6	38.6	80.6		13.4	4.7	38.6		27	13.4	54.3	
**Donor-recipient sex match**				0.81				0.66				0.17				0.42
Same sex (n=33)	57.2	38.6	75.8		44.6	26	63.2		45.7	30.3	68.8		9.8	3.3	28.6	
M to F (n=11)	47.1	14.6	79.6		47.7	15.2	80.2		30.1	11.5	79		22.2	6.7	73.1	
F to M (n=23)	58	33.5	82.5		54.4	30.5	78.3		18.5	7.7	44.8		27.1	12.1	60.7	

ALL, acute lymphoblastic leukemia; BM, bone marrow; CI-NRM, cumulative incidence of non-relapse mortality; CIR, cumulative incidence of relapse; CNS, central nervous system; CR1, first complete remission; CR2, second complete remission; EFS, event-free survival; F, female M, male; OS, overall survival; PB, peripheral blood; Ph-positive, Philadelphia chromosome-positive; PTC; posttransplant cyclophosphamide; TBI, total body irradiation. *Hypodiploidy ALL and t(4;11). Bold values means values with statistical significance as defined in statistical analysis section.

### Relapse and non-relapse related mortalities

**Figure 2:**
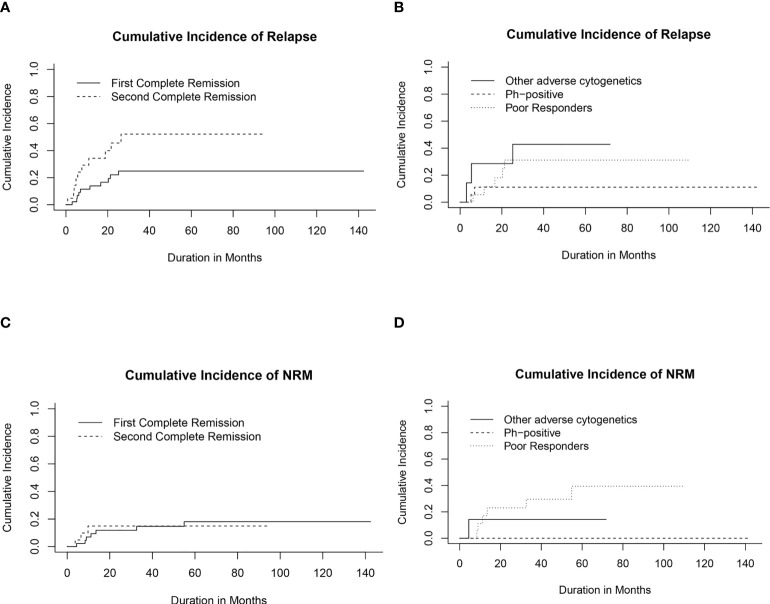
Cumulative incidence of relapse (CIR) and non-relapse mortality (CI-NRM). **(A)** CIR for transplanted patients in first (CR1) and second (CR2) complete remission. **(B)** CIR in relation to high-risk features in CR1 transplants. **(C)** CI-NRM for transplanted patients in first (CR1) and second (CR2) complete remission. **(D)** CI-NRM in relation to high-risk features in CR1 transplants.

Twenty patients (29.8%) relapsed post-HSCT. The median time to relapse post-HSCT was 7.3 months (range, 0.9-26.4 months). Ten of the patients who suffered relapses (21.7%) were in CR1 group. They included five patients who had a poor induction response, two with Ph-positive ALL, two with hypodiploid cytogenetics and one with t(4, 11). The 5-year CIR for the whole cohort was 33.5% (95% CI, 21.7%-45.8%) with statistically significant lower CIR in CR1 transplants (24.8%, 95% CI, 12.6%-39.2%) compared with CR2 (52.1%, 95% CI, 26.4%-72.7%), p= 0.02 (**Table 4**; **Figure 2A**).

Among patients transplanted in CR1, the lowest CIR was recorded for patients with Ph-positive ALL compared to other CR1 transplants (**Figure 2B**).

Ten out of 21 patients (47.6%) who had been transplanted in CR2 eventually relapsed. Importantly, the time period to the first pretransplant relapse and the site of relapse in CR2 transplants did not appear to significantly influence relapse probabilities post-HSCT (**Supplementary Figures 1A, B** **)**.

Ten patients died in remission with a median time from date of transplant of 9.4 months (range, 3.8- 54.9 months). Six died of refractory chronic GVHD complications; 2 of septic shock and 2 of diffuse alveolar hemorrhage. The 5-year CI-NRM was 17.3% (95% CI, 8.67%-28.6%) with no statistically significant differences between patients transplanted in CR1 and CR2 (**Figure 2C**). Among patients who had undergone transplant in CR1, none of those with Ph-positive ALL experienced NRM, while the highest CI-NRM was noted in patients with a poor response to induction therapy (39.4%, 95% CI, 13.5%-64.8%, p=0.02) (**Figure 2D**).

## Discussion

Despite limited resources as a center in a developing country, we were able to include in this study a group of homogeneously treated ALL patients who had received a modified risk adapted protocol (based on the St. Jude TXV-ALL protocol) and had undergone HSCT from MSDs receiving MAC regimen with a pretransplant confirmed negative MRD (<0.01%).

In this study the high CIR noted after CR2 transplants resulted in a lower 5-year EFS (49%) for the whole cohort compared to studies by Peters et al. (15) and Yanir et al. (18), who reported a 4-year EFS of 71% and 5-year EFS of 63%, and CIR of 22% and 20%, respectively.

Conducting an accurate comparison of outcomes of different studies is difficult as many factors contribute to post-HSCT disease outcome including; selection criteria for high-risk groups, types of donor and sources of stem cells, and types of conditioning regimens. We observed better OS, EFS and CIR in CR1 compared to CR2 transplants. Peters et al. (15) had reported similarly better outcomes in CR1 transplants from MSD with a 4-year EFS and relapse incidence of 0.79 ± 0.06 and 0.17 ± 0.05 compared to 0.62 ± 0.07 and 0.32 ± 0.07 for subsequent remissions with no differences observed based on donor type (MSD and MUD) or stem cell sources (BMSCs and PBSCs).

A report from the AIEOP (Italian Pediatric Hematology-Oncology Association) registry (19) described a 10-year OS, DFS and CIR in CR1 transplanted patients of 63.4% (95% CI, 57-70), 61% (95% CI, 54-68) and 24% (95% CI, 19-30), respectively, their results being comparable to our 5-year probabilities. Marshall et al. (20) reported in CR1 transplants, a comparably higher 5-year OS of 82.7% ( ± 5.3) and EFS of 73.3% ( ± 7.8).

Fagioli et al. (19) described an inferior transplant DFS in patients 10-14 years at diagnosis compared with younger patients (RR,1.66; 95% CI, 1.01-2.74; P=0.045), which they attributed to higher transplant related mortalities. In the current study, higher CIR were observed in patients younger than 10 years at diagnosis compared with older patients (p=0.007). This may be attributed to the higher percentage of CR2 transplants conducted for these younger patients compared with the older ones (38% vs 20%, respectively). This higher CIR did not however reflect statistically on differences in EFS between both groups as it was counterbalanced by the lower NRM observed in our younger patients.

Early TRM was not observed among our patients. Nevertheless, the 5-year CI-NRM (18%; 95% CI, 7.7%-32%) was higher in this study compared to a BFM trial that reported a NRM of 10% from MUD-HSCT and 3% from MSD-HSCT. The disparity in results could be attributed to the high prevalence of cGVHD in our patients (15). Chronic GVHD remains a major cause of NRM after allogeneic HSCT and for financial reasons, we are to date unable to provide patients with the latest modalities used to overcome refractory cGVHD, including targeted therapies and extracorporeal photopheresis (21).

Total body irradiation-based regimens were previously studied and shown to be effective preparative regimens that are nevertheless coupled with long-term side effects (22). Due to the small numbers of patients who received busulphan-based regimen (n=14), no further subgroup analyses were possible. However, for the whole cohort, no statistically significant difference was observed in survival rates or relapse probability between patients transplanted using TBI- and busulphan-based regimens. These results stand in contrast to those of Bunin et al. (23) and Davies et al. (24), who reported significantly inferior 3-year EFS in pediatric ALL patients who had received busulphan-based compared to TBI-based conditioning regimens. In a recent multicenter randomized study of over 400 pediatric patients with ALL undergoing HSCT, higher survival and lower risk of relapse have been observed following TBI-based regimen compared to chemotherapy conditioning regimens with fludarabine, thiotepa, and either busulfan or treosulfan with two-year CIR of 0.12 (95% CI, 0.08 to 0.17; P <.0001) and 0.33 (95% CI, 0.25 to 0.40), respectively (25).

We used BM more frequently than PB as a stem cell source. In this study, there were no notable differences in survival rates or relapse probabilities with respect to stem cell sources in either CR1 or CR2 transplants. Simonin et al. (26) reported higher OS with BM compared with PB transplantation (67% vs 62%; P=0.0004), as well as a better leukemia-free survival (59% vs 54%; P=0.0007).

The relationship between different stem cell sources and the risk of developing either acute or chronic GVHD is an ongoing controversy. Some studies reported rates of grade II-IV aGVHD after allogeneic PBSC transplantations that were comparable to or slightly greater than those observed after BMSC transplantations (27–29). On the other hand, Schmitz et al. (30) reported significantly higher rates of aGVHD of grades II-IV associated with PBSC transplantation compared to recipients of BMSC (52% vs 39%; odds ratio, 1.74; 95% CI, 1.12-2.69; P=.013). In the current study, more aGVHD occurred among recipients of BMSCs, a result that may be attributable to the use of ATG or PTC as GVHD prophylaxis in 11/25 (44%) of PBSC recipients. Furthermore, there was a higher occurrence of cGVHD among patients who had received PBSC transplants and MTX plus CSA for GVHD prophylaxis (43%) than when combined with ATG or PTC (0%). Similarly, the EBMT confirmed a higher the risk for cGVHD with PB source plus MTX and CSA compared to BM in MSD transplant (26). Russell et al. (31) has noticed lower TRM and cGVHD in patients with hematological malignancies after the use of ATG in recipients transplanted from related-matched donors. Jacoby et al. (32) reported that no aGVHD or cGVHD occurred in patients who had received PTC in a related-matched donor transplant setup. Given the previous results, large prospective randomized pediatric studies are needed to compare the use of ATG and PTC for GVHD prophylaxis in MSD transplants and to determine their impact on the incidence of acute and chronic GVHD.

Our institutional policy was to transplant Ph-positive ALL in CR1, if they have MSD even if they achieved negative MRD by flowcytometry at the end of induction. Notably, an excellent 5-year CIR (11%; 95% CI, 1.74-30.4%) was observed in this group of patients with statistically better EFS (88.9%; 95% CI, 74.4-100%) and CI-NRM (0%) compared with other high-risk patients. Sharathkumar et al. (33) reported a 4-year EFS of 53 ± 15% in Ph-positive ALL transplanted from HLA-matched related donor and MUD. The authors recounted the inferior outcome for patients who had suffered relapse and the difficulty to achieve a sustained second remission even with HSCT. Other studies conducted on pediatric patients reported a DFS of > 70% (34–36). The AIEOP group enrolled 43 patients with t (9, 22) in their study and reported a 10-year DFS of 60%; a result comparable to that of their CR1 transplants for patients with Ph-negative ALL (19). On the other hand, the COG and other study groups continued to recommend postponing HSCT for Ph-positive ALL in first remission for patients who achieve an early MRD negative results at the end of induction. This recommendation was based on the finding of comparable EFS rates after the treatment with TKI combined with intensive chemotherapy versus conducting matched-family donor HSCT in CR1 (10, 37). The role of MUD-HSCT in CR1 versus chemotherapy in Ph-positive ALL patients is a point of debate and still no proof of significant privilege for MUD-HSCT over chemotherapy (38).

Induction failure and high MRD levels post-induction therapy are indicators of poor post-HSCT outcome. Amongst our CR1 transplanted patients, the lowest OS (40%) and EFS (29.5%) were observed in those transplanted after a poor response to induction therapy. This modest outcome, despite the fact that patients had been transplanted with an MRD <0.01%, suggests the need for more sensitive methods, besides flowcytometry, to more ‘deeply’ detect MRD (e.g. PCR- and NGS-MRD methods). A better determination of the depth of MRD would allow for better decision making with regards to the need for further intensified chemotherapy or immunotherapy before undergoing HSCT (39).

In a group of patients with IF, Schrappe et al. (4) reported a better transplant outcome for T-cell ALL (5-year EFS of 40–45%) compared to B-cell ALL in consistency with the findings reported by Conter et al. (40). We observed in 10 transplanted patients with IF, six T-cell ALL patients had sustainable remission post-HSCT and only one them died of cGVHD complications, while 3 out of 4 B-cell ALL had relapsed and died.

Allogeneic-HSCT has a definitive curative role in many hematological malignancies. However, in developing countries with limited resources many limitations exist that reflect on the management and outcome of HSCT (41). The importance of this study lies in the fact that it describes the practice and the outcome of HSCT for pediatric ALL patients in a developing country. The retrospective nature of this study from a single-center is considered a limitation. The small number of CR2 transplants made the interpretation of many factors associated with their outcome limited. Lastly, another source of potential weakness could be related to the policy of transplanting Ph-positive ALL, regardless the MRD induction response.

We did not aim by this study to compare the outcome of ALL with high-risk features treated with chemotherapy alone with those who had undergone transplant but still our data suggested that allogeneic-HSCT could improve the outcome in a subset of ALL patients, especially those transplanted in their CR1. Transplant from MSDs is a curative option for Ph-positive ALL without an increased risk of non-relapse mortality. Larger pediatric studies are still needed to evaluate the outcome of TKI in combination with chemotherapy versus CR1 transplant in Ph-positive ALL. These lower survival rates and high relapse probabilities observed in patients who failed to have a good induction response, and in patients who suffered relapses, mandate further refinement of the pretransplant selection criteria, including better assessment of the depth of MRD response before transplant by more sensitive methods (e.g. PCR-MRD) and applying mutational studies by next generation sequencing in order to improve the outcome by immune-based therapeutic approaches.

## Data availability statement

The original contributions presented in the study are included in the article/**Supplementary Material**. Further inquiries can be directed to the corresponding author.

## Ethics statement

The studies involving human participants were reviewed and approved by Children’s Cancer Hospital Egypt (CCHE-57357) Scientific Medical Advisory Board. Written informed consent to participate in this study was provided by the participants’ legal guardian/next of kin.

## Author contributions

Conceptualization, MH and HH. methodology, MH, HH and AE. software, KA. validation, IS and AE. formal analysis, MH, IS, and KA. investigation, DY, SS, NH and NE. writing—original draft preparation, MH and HH. writing—review and editing, MH HH, IS and AE. visualization, MH, HH and KA. supervision, AE. project administration, MH, HH and AE. All authors contributed to the article and approved the submitted version.

## Conflict of interest

The authors declare that the research was conducted in the absence of any commercial or financial relationships that could be construed as a potential conflict of interest.

## Publisher’s note

All claims expressed in this article are solely those of the authors and do not necessarily represent those of their affiliated organizations, or those of the publisher, the editors and the reviewers. Any product that may be evaluated in this article, or claim that may be made by its manufacturer, is not guaranteed or endorsed by the publisher.

## Supplementary material

Supplementary Figure 1 Cumulative incidence of relapse (CIR) for transplanted patients in CR2 in relation to **(A)** initial time of pretransplant relapse and **(B)** initial site of pretransplant relapse.
