# Supplementary material for: Hematopoietic stem cell transplantation from HLA-matched sibling donors in children with acute lymphoblastic leukemia: A report from the Children’s Cancer Hospital Egypt
Source: Front Oncol. 2022 Sep 27;12:983220. doi: 10.3389/fonc.2022.983220 (PMC9551213; doi:10.3389/fonc.2022.983220)
Supplement: Supplementary file 1 [file DataSheet_1.docx]

**Supplementary Table 1:** Risk of developing aGVHD and cGVHD in relation to different transplant-related factors

**Supplementary Table 2:** Acute and chronic GVHD in relation to different transplant-related variables

| **Supplementary** **Table 1:** Risk of developing aGVHD and cGVHD in relation to different transplant-related factors | | | | | | | | |
| --- | --- | --- | --- | --- | --- | --- | --- | --- |
|  | Acute GVHD | | | | Chronic GVHD | | | |
| Characteristics | odds ratio | 95% Confidence Intervals limits | | P value | odds ratio | 95% Confidence Intervals limits | | P value |
|  |  | Lower | Upper |  |  | Lower | Upper |  |
| Donor Gender (F vs M) | 2.5 | 0.847 | 7.245 | 0.09 | 1.2 | 0.405 | 3.669 | 0.72 |
|  |  |  |  |  |  |  |  |  |
| Donor-recipient Sex (different vs Same) | 1.275 | 0.446 | 3.646 | 0.65 | 1.548 | 0.508 | 4.714 | 0.44 |
|  |  |  |  |  |  |  |  |  |
| CR1 vs CR2 | 0.788 | 0.259 | 2.394 | 0.67 | 2.625 | 0.664 | 10.373 | 0.16 |
|  |  |  |  |  |  |  |  |  |
| Conditioning (TBI vs Non-TBI) | 1.731 | 0.427 | 7.027 | 0.44 | 5.622 | 0.677 | 46.685 | 0.11 |
|  |  |  |  |  |  |  |  |  |
| BM vs PB | 4.987 | 1.287 | 19.323 | **0.02** | 1.124 | 0.357 | 3.3538 | 0.84 |
|  |  |  |  |  |  |  |  |  |
| Age at transplant (<=12 years vs >12 years) | 0.281 | 0.094 | 0.841 | **0.02** | 0.672 | 0.216 | 2.089 | 0.49 |

Abbreviations: aGVHD, acute graft versus host disease, cGVHD, chronic graft versus host disease; CR1, first complete remission; CR2, second complete remission; BM, bone marrow; F, female; M, male; PB, peripheral blood; TBI, total body irradiation.

| **Supplementary** **Table 2:** Acute and chronic GVHD in relation to different transplant-related variables | | | | | |
| --- | --- | --- | --- | --- | --- |
| **Characteristics** | **Number of patients** | **Acute GVHD (%)** | **P** | **Chronic GVHD (%)** | **P** |
| Donor Gender |  |  | 0.048 |  | 0.725 |
| Female | 33 | 13 (39.4) |  | 9 (27.3) |  |
| Male | 34 | 6 (17.7) |  | 8 (23.5) |  |
| Donor/Recipient Sex Match |  |  | 0.25 |  | 0.757 |
| Female to Male | 22 | 9 (40.9) |  | 6 (27.3) |  |
| Male to Female | 12 | 2 (16.7) |  | 4 (33.3) |  |
| Same Sex | 33 | 8 (24.2) |  | 7 (21.2) |  |
| Disease Status |  |  | 0.98 |  | 0.159 |
| CR1 | 46 | 13 (28.3) |  | 14 (30.4) |  |
| CR2 | 21 | 6 (28.6) |  | 3 (14.3) |  |
| Age at transplant |  |  | **0.01** |  | 0.491 |
| ≤ 12 years | 44 | 8 (18.2) |  | 10 (22.7) |  |
| > 12 years | 23 | 11 (47.8) |  | 7 (30.4) |  |
| Conditioning Regimen |  |  | 0.19 |  | 0.078 |
| TBI based | 53 | 17 (32.1) |  | 16 (30.2) |  |
| Non TBI based | 14 | 2 (14.3) |  | 1(7.1) |  |
| Graft Source |  |  | **0.02** |  | 0.842 |
| BM | 42 | 16 (38.1) |  | 11 (26.2) |  |
| PB | 25 | 3 (12) |  | 6 (24) |  |
| GVHD prophylaxis |  |  | 0.13 |  | 0.071 |
| CSA+MTX | 54 | 18 (33.3) |  | 17 (31.5) |  |
| CSA+MTX+ATG | 8 | 0 (0) |  | 0 (0) |  |
| CSA+MTX+PTC | 5 | 1 (20) |  | 0 (0) |  |

Abbreviations: ATG, Anti-thymocyte globulin; BM, bone marrow; CR1, first complete remission; CR2, second complete remission; CSA, cyclosporine; GVHD, graft versus host disease; MTX, methotrexate; PB, peripheral blood; PTC; posttransplant cyclophosphamide; TBI, total body irradiation.
