# Supplementary figures and images for: Hematopoietic stem cell transplantation from HLA-matched sibling donors in children with acute lymphoblastic leukemia: A report from the Children’s Cancer Hospital Egypt
Source: Front Oncol. 2022 Sep 27;12:983220. doi: 10.3389/fonc.2022.983220 (PMC9551213; doi:10.3389/fonc.2022.983220)

## Cumulative Incidence of Relapse

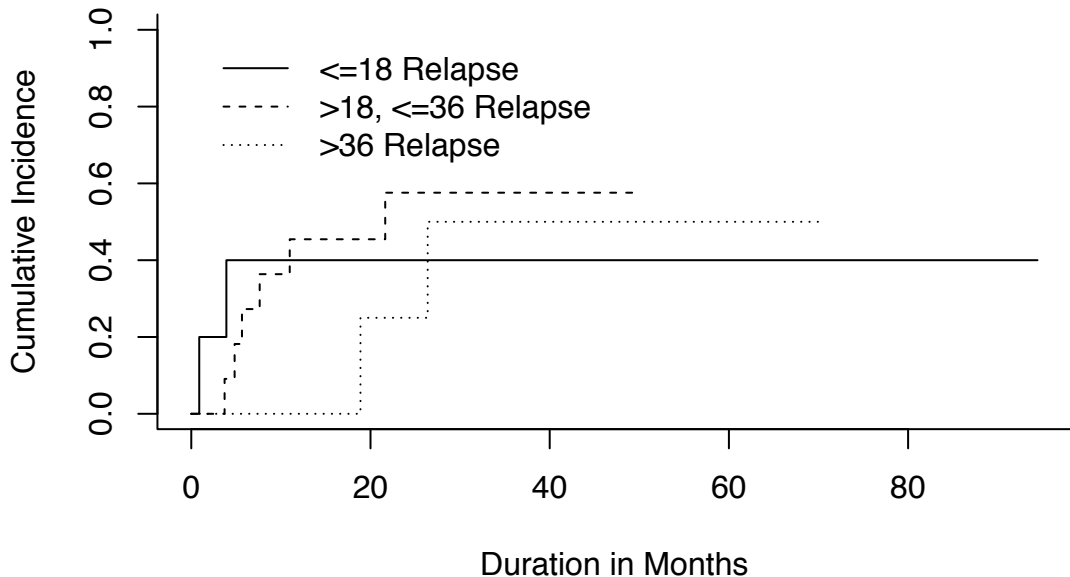

Supplement: Supplementary Figure 1 — Cumulative incidence of relapse (CIR) for transplanted patients in CR2 in relation to (A) initial time of pretransplant relapse and (B) initial site of pretransplant relapse. [file DataSheet_2.zip › Supplementary Fig 1 A.pdf]

## Cumulative Incidence of Relapse

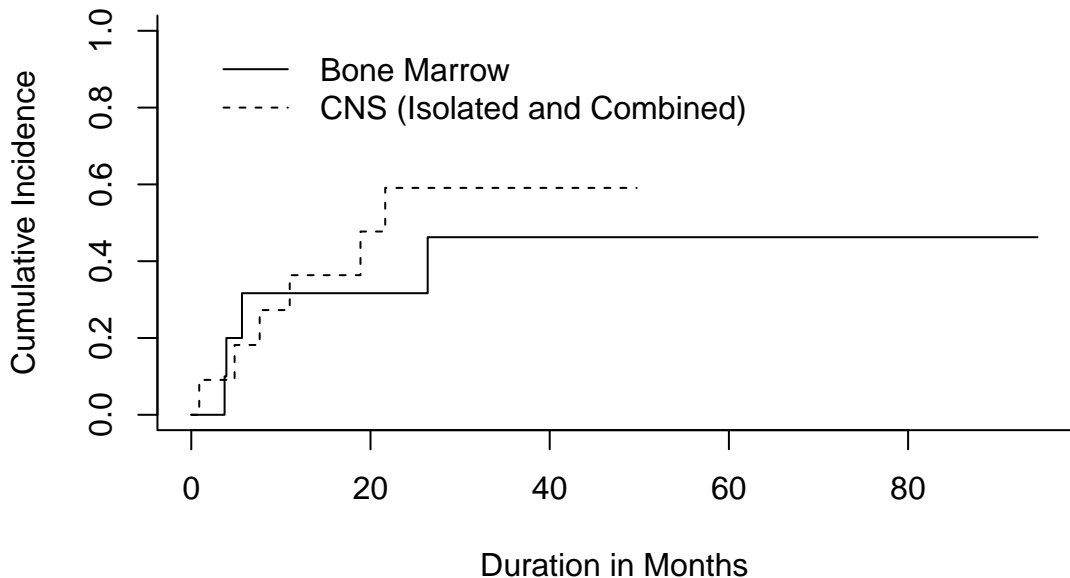

Supplement: Supplementary Figure 1 — Cumulative incidence of relapse (CIR) for transplanted patients in CR2 in relation to (A) initial time of pretransplant relapse and (B) initial site of pretransplant relapse. [file DataSheet_2.zip › Supplementary Fig 1B.pdf]
